# Supplementary material for: Are carbon emissions trading and green financial instruments synergistic? -Comprehensive quantitative research based on content analysis
Source: PLoS One. 2024 Mar 7;19(3):e0298601. doi: 10.1371/journal.pone.0298601 (PMC10919601; doi:10.1371/journal.pone.0298601)
Supplement: S1 Data — (ZIP) [file pone.0298601.s001.zip › Data/地级市Z_F工作报告.zip!/╜¡╬≈/╨┬╙α╩╨/└┤╫╘╣1⁄2╓┌║┼í╛┬φ┐╦╩2╛▌═°í┐_2005.docx]

各位代表：

现在，我代表市人民政府向大会作政府工作报告，请予审议，并请市政协委员和其他列席会议的同志提出意见。

一、２００４年的工作回顾

过去的一年，全市上下深入贯彻党的十六届三中、四中全会精神，以加快区域中心城市建设为主题，围绕“一条主线、五篇文章”的总体发展思路，与时俱进，开拓创新，圆满完成了市六届人大二次会议确定的目标任务。

———以工业化为战略核心，国民经济快速发展，主要经济指标提前一年完成和超额完成了“十五”计划。全市完成生产总值１３４亿元，比上年增长１６．５％，比“十五”计划预期目标１１４．５亿元多１９．５亿元；完成全社会固定资产投资６８．０亿元，增长３０．８％，四年累计完成投资１８５．２亿元，比“十五”计划预期目标１００亿元多８５．２亿元；完成社会消费品零售总额３９．５亿元，增长１４．５％。全市实现财政总收入１８．１８亿元，增长２５．０％，其中地方财政收入７．５４亿元，增长２０．４％，分别比“十五”计划预期目标值的１０亿元、５．７８亿元多８．１８亿元和１．７６亿元。金融机构各项存款余额１３６．８６亿元，贷款余额９２．６７亿元，分别增长１９．６％和１８．１％。工业主导型经济增长格局进一步突出，第二产业增加值占生产总值的比重提高２．５个百分点。全市规模以上工业增加值４６．０亿元，销售收入１６６．２亿元，利税总额１１．８亿元，分别比２００１年增长２．１０倍、１．５４倍和１．１５倍，提前一个月完成三年翻番目标。新钢公司销售收入首次突破百亿大关，成为全省首个单个企业销售收入过１００亿元的企业。高新开发区继２００３年荣获全省招商引资十强园区之后，晋升为全省十大重点园区之一。

———以项目建设为主抓手，一批重点项目相继实施，经济发展后劲进一步增强。大力实施项目带动战略，全年实施重点项目７３项，项目投资总额９７．８亿元。其中，实施投资千万元以上工业技改项目２２项，实施重点农林水项目５项，实施交通、电力和城市基础设施建设项目２２项。目前，３２个重点项目已经完工或完成年度投资计划，３５个项目开工在建，６个重点项目即将开工。随着新钢公司中厚板、国电公司５００千伏变电站、分宜电厂２０万千瓦机组技改等１５个投资过亿元的重大工业项目的实施，随着沪瑞高速公路的建成通车和浙赣铁路电气化改造的加快，我市经济发展后劲和区位优势得到进一步提升。

———以“三城”创建为目标，城市功能日趋完善，城市承载辐射能力进一步提升。积极开展“三城”创建活动，明确提出了２００５年争创国家园林城市、２００６年争创国家卫生城市、２００７年争创全国文明城市的目标，推动城镇建设纵深展开。整治了违章建筑，重点对四眼井等居民小区及城市背街小巷实施了综合整治，城市管理进一步规范，市容市貌及环境卫生有了明显改观。组织修编了城市总体规划，完成了孔目江整治开发规划设计。着力抓好一批重点工程。铁路二通道正在建设，市艺术中心交付使用，渝州大桥、新欣大道建成通车，一批城市道路相继开工，主城区新增道路２．４５平方公里。抱石名园等一批商贸旅游重大项目正在抓紧完成拆迁安置工作。一批现代化住宅小区相继建成。以河下小康红旗镇为重点，文明村镇建？？？加速推进。１０个中心镇进行了以“三位一体”改造为重点的基础建设，村村通水泥公路三年任务提前一年基本完成。

———以改革开放为动力，加速推进体制机制创新，开放带动型经济增长格局进一步形成。事业单位改革试点全面启动，国企改革继续深入。长林集团债转股、市床上用品厂和江西电工厂改制工作顺利推进，前卫公司、市通用机械厂依法破产终结。市属五大流通领域企业改制工作全面推进，提前一年基本完成国有商业企业改制任务。招商引资呈现大项目多、工业项目多、高科技含量项目多的喜人局面。全市新批国外境外合同项目２８个，实际利用国外境外资金５１５７万美元，实际引进国内省外资金４４．１亿元，分别增长３８．８％和２６．７％。飞宇工业园、春龙反光材料工业园等２１个投资过亿元的大型项目相继落户我市，初步形成了以大项目为龙头、产业配套延伸的集聚效应。外贸进出口一举扭转多年持续下滑趋势。全市完成外贸进出口总额１４３９１万美元，其中外贸出口２２７１万美元，分别增长２７６．８％和７６．５％。

———以贯彻落实党的“三农”政策为纲，认真抓好粮食生产，农业基础地位进一步加强。切实加大惠农、支农力度，全面落实了粮食直补、良种补贴、粮食最低收购价等各项惠农政策。农村减负工作落到实处，农民人均负担由上年的６１．８１元调减为３５．２３元。粮食生产呈现“一增三超”的喜人局面。即全市粮食面积达１３９万亩，比上年增长１９．０％；粮食单产、总产和收入实现超历史，全市农民人均可从粮食生产中增收３０３．４元。农业产业化经营加快推进，龙头企业带动力不断增强。全市龙头企业已发展到８１家，其中省市级龙头企业发展到１６家。农田水利基本建设得到加强，启动了病险水库除险加固，农业生产条件得到进一步改善。

———以改善人民生活为根本，加快构建城乡一体的就业体系，社会保障水平进一步提高。城镇居民人均可支配收入７８６９元，增长１１．０％。农民人均纯收入３４２４．７元，增长２０．２％。城乡居民储蓄存款余额９３．７亿元，增长１８．６％。就业再就业各项任务全面完成。全市新增城镇从业人员２１６３２人，国有企业下岗职工再就业６８０９人，安置困难群体就业１２７８人，省外劳务输出１６２７５０人。“两个确保”全部到位，离退休人员养老金发放率及社会化发放率、下岗职工基本生活费发放率及社会保险费代缴率均为１００％。城镇低保对象基本实现应保尽保。农村困难群众得到有效救助，市、县（区）两级配套资金全部落实到位，确保了常补对象人均年救助２００元以上。

———以经济社会统筹发展为方略，民主法制建设得到加强，各项社会事业进一步发展。组织开展了全市经济普查。科技项目申报工作取得明显成效，申报列入省级重点新产品计划项目３４项，列入国家级项目４个。省市共建科普示范城工程正式启动。基础教育、高等教育稳步发展，农村中小学危房改造和远程教育扎实推进，优质高中建设走在全省前列。普通高考成绩喜人，二本上线率居全省第一。职业教育、民办教育位居全省首位，一批重点院校办学规模进一步扩大。无线广播电视基本覆盖全市农村，一批优秀广电、文艺作品在全国、全省获奖，成功举办了纪念傅抱石诞辰１００周年系列活动。《欢天喜地七仙女》、《会说话的风筝》等一批影视剧目在我市开拍，新余对外宣传推介力度进一步加大。 维护食品、药品安全和公共卫生安全得到加强，农村新型合作医疗试点工作走在全省前列。成功举办了全市第五届运动会，承办第五届全国农运会乒乓球比赛被评为全国最佳赛区。国防动员和民兵预备役得到加强，军事斗争准备充分。质量技术监督工作荣获全国先进，计划生育工作综合评比排名全省第一，市人口计生委被评为全国计生系统作风建设先进单位。妇儿、信访、环保工作保持全省领先。民主法制建设全面进步，反腐倡廉和政府自身建设明显加强。我市成为全省唯一跻身中国城市竞争力排行榜“诚信政府”前３０名的设区市和全省唯一荣获２００４年全国地方电子政务应用调查综合奖的单位。创建“平安新余”得到加强，社会治安保持稳定，第１１次荣获全省社会治安综合治理先进市。金融、保险、银监、工商、税务、公路、稽征、邮政、通讯、石油、烟草、盐业、医药、气象、地震、无线电、新闻出版等各项事业实现新发展，旅游、审计、统计、物价、人事、编制、外事、侨务、民族宗教、档案、老龄、残联、地方志、驻外机构等各项工作都有新进步。

各位代表，去年的经济工作是在国家加大宏观调控力度下展开的。一年来，我们坚持以科学发展观为指导，认真贯彻国家加强宏观调控的各项政策，严格落实省委、省政府“三个不搞”的有关规定，全面组织开展固定资产投资项目清查；落实“六清理”，实施“六整顿”，积极推进土地市场秩序整治，全面开展基本农田保护检查，严格执行耕地占补平衡制度，确保了耕地动态平衡。在克服资金紧缺、煤电运紧张、原材料涨价等不利因素的影响下，成功实现了“两个确保”的目标，确保了国家宏观政策的落实到位，确保了经济的快速发展。这是全市人民在省委、省政府和市委的正确领导下开拓创新、团结拼搏的结果，是人大依法监督、政协民主监督和积极参与支持的结果，是驻市中央省属单位、部队、武警、公安民警、各民主党派、工商联、人民团体、离退休老同志和台湾同胞、港澳同胞、华人华侨大力支持的结果。在此，我代表市人民政府，向所有关心、支持新余发展的同志们、朋友们表示衷心的感谢和崇高的敬意！

同时，我们也清醒地看到发展中存在的困难和问题：支柱产业单一，经济结构不合理，非公有制经济发展相对滞缓，工业发展瓶颈制约日益突出；就业再就业压力加大，社会保障特别是困难群体生活保障任务越来越重，城市化、工业化进程中居民拆迁、征地补偿、失地农民转岗就业和养老、社保矛盾更加凸显，社会不稳定因素依然存在；机关作风和政府效能亟待改进，投资环境有待进一步优化。对此，我们将采取有力措施，逐步加以解决。

各位代表，过去的一年，我们朝着率先建设全面小康社会的宏伟目标迈出了坚实的一步。但是，与全国、全省先进相比，我们还有很大的差距。站在新的更高起点上，我们要进一步强化危机意识，抢抓机遇，积极进取，全面开创我市改革开放和现代化建设的新局面！

二、２００５年的主要目标和工作重点

今年是 “十五”计划的最后一年，也是巩固宏观调控成果、保持经济社会良好发展态势的关键一年。做好今年的工作，意义十分重大。今年经济社会发展工作的指导思想是：深入贯彻党的十六届三中、四中全会精神和省委十一届八次全会精神，坚持以科学发展观为指？？？，坚决落实中央宏观调控政策；按照“一条主线，五篇文章”的总体发展思路，以培育优势产业集群为核心，以“十大工程”建设为主抓手，坚定不移地推进改革开放，坚定不移地推进工业化、城市化和农业产业化；大力开展城市创建活动，高标准建设现代区域中心城市；坚持以人为本，切实关心和解决好人民群众的实际问题，加快构建和谐平安新余，统筹推进城乡经济和社会事业快速协调发展。

经济社会发展的主要预期目标是：全市生产总值１５４亿元，增长１５％。财政总收入２１．１亿元，其中地方财政收入８．７５亿元，分别增长１６％。全社会固定资产投资８８．４亿元，增长３０％；社会消费品零售总额４４．６亿元，增长１３％。城镇居民人均可支配收入８６５６元，农民人均纯收入３７６７元，分别增长１０％。人口自然增长率控制在省定目标以内。

完成上述目标，重点要做好以下工作：

（一）全面深化以市场为取向的各项改革，加快构建大开放、大发展的经济运行格局

坚定不移地实施大开放主战略，推动开放型经济发展跨入新阶段。招商引资工作坚持“四个突出”，突出港台、江浙等重点地区、重点客商展开招商；突出工业园区的招商主战场地位，继续抓好飞宇、华源、春龙等已落户企业的跟踪服务；突出大项目的带动作用，积极开展工业项目招商年活动；突出投资环境的优化改善，整治和规范市场经济秩序，优化金融生态环境，积极推进“信用新余”建设，着力打造“诚信政府”、“诚信企业”、“诚信社会”，加快建成海关、商检机构，严格落实项目代办制、首问责任制、责任追究制等制度，努力营造全社会亲商、重商、安商、富商的浓厚氛围。同时坚持“三个创新”，创新招商主体、招商方式和招商机制，组建离岸招商公司，大力推行代理招商、以商招商，积极聘请一批中介机构、国内外知名企业、社会知名人士委托招商，培育发展一批招商经纪人。切实加强招商队伍建设，充分发挥驻外机构的招商作用，加强招商引资项目库建设。着力构建“三外”并举的发展格局，发展外经，拓展外贸，努力做到对外经济技术合作有新突破。确保全年实际利用国外境外资金６０００万美元，实际引进国内省外资金４８亿元人民币，外贸进出口总额突破２亿美元。

全面深化以市场为取向的各项改革，加快建立比较完善的社会主义市场经济体制。积极组织实施国有集体企业两年改制攻坚战役。今年，全面完成市属工商企业的改组改制；争取通过两年努力，完成农口企业、城建交通企业的改革。切实抓好国有资产管理体制改革。健全国有资产三级营运架构，建立责、权、利相统一，管人、管事和管资产相结合的国有资产管理体制。严格控制土地一级市场，完善土地储备中心职能，强化土地储备。发挥信用担保中心职能，补充担保资金，不断增强担保融资能力；组建危旧房拆迁改造公司，积极做好被拆迁房屋的土地抵押贷款和市场运作；充分发挥城投公司和国资经营公司的作用，把政府有效资产授权给城投公司，把可经营的国有资产授权给国资经营公司，实行统一经营、管理。今年，先将机关、事业单位所属经营性资产全部授权给国资经营公司统一经营。按照整体推进、配套改革的要求，全面完成事业单位改革任务。深化乡镇机构改革，改革财税管理体制。全面推行综合预算，加快建立公共财政管理体制，积极推进？？？乡财县代管”改革试点；采劝核定基数、超收分成”的办法，推行城区税收统一征管。积极支持金融机构改革发展，全力支持组建农村合作银行。深化林业产权制度改革，完善粮食流通体制改革，加快殡葬事业改革步伐。

放手发展民营经济，加快构建以非公有制经济为主导的经济增长新格局。全面落实加快民营、中小企业发展的“十条政策”，实行民营企业新、扩、改项目在征地、税收等方面与外资企业的同等待遇。设立中小企业发展专项资金，建设民营中小企业孵化基地。采取领导挂点帮扶、银行授信、专家科技联姻等办法，在开发新产品、技改贴息、跟踪服务等方面对良山钢管、永盛工贸等民营企业前１０强予以重点扶持；优选一批企业进入上市辅导期，加快培育一批销售收入超亿元的民营企业。

（二）大力组织实施工业年销售收入５００亿元工程，不断增强区域中心城市的核心竞争力

坚持以工业为立市兴城之本，按照“做强一个龙头，做大三个板块”的要求，以全省工业现场会在我市召开为契机，做大工业总量，优化产业结构，大力实施工业销售收入５００亿元工程，力争通过工业三年决战，到２００７年，实现规模以上工业销售收入５００亿元目标，其中新钢公司２００亿元，高新开发区１５０亿元，分宜县５０亿元，渝水区５０亿元，仙女湖区１０亿元，以及除新钢公司以外的中央、省市属企业４０亿元。

把支持中央省属企业作为加快工业发展的重要举措，坚定不移地支持驻市企业做大做优做强。协助新钢公司抓好中厚板、６＃高炉维修等重点技改项目，支持新钢公司实施“２３４８”第三期技改工程，力争通过３～５年努力，尽快达到总资产２００亿元、年销售收入３００亿元、年税利４０亿元、年产钢８００万吨的目标；配合抓好分电公司３０万千瓦机组和新电公司２×６０万千瓦装机申报立项工作，支持赣西供电局在高新区和分宜县各上一个２２万伏变电站，支持花矿矸石电厂２×５万千瓦机组扩建工程、花矿和杨桥煤矿两个３０万吨矿井建设，支持省煤炭集团在我市设立矿山救护队和煤炭物资配送中心。主动承担企业办社会职能，配合做好江西二化依法破产和江西钢丝厂债转股工作，积极促成江西锻压厂、煤矿电机厂等企业改制重组。完成铁路二通道建设，争取实施袁河航道改造工程，为驻市企业加快发展创造条件。

着力扶持一批成长性好的骨干企业，积极优选除新钢外的１００户重点企业实行重点扶持，加快培育一批年销售收入超１０亿元的重点骨干企业。实施质量兴市和名牌战略，发展高效、低耗产业，大力组织实施４０项总投资１３４．８亿元的重点技改项目，推动工业技术跨越式进步。年内，完成工业技改投入３５亿元，重点支持分宜海螺再上一条日产５０００吨水泥熟料生产线，支持华源纺织公司兴建高档服装面料和高档丝光烧毛纱线两条生产线，支持飞宇、春龙、华源地毯、中铁利达和香港财富加快建设，积极促成彬晖服装、流星实业、王龙化工、华通电工、可视电话等工业项目开工建设。加快标准化中药材基地建设，积极推动以新余制药、新和药业、欧氏药业和青春康源为龙头的医药产业迅速成长。

大力加强园区建设，加快构建工业经济腾飞的新高地。坚持举全市之力支持高新区发展，多方筹资搞好园区基础设施建设。抓好已签约洽谈项目的进资履约，加快已入？？？进园工业项目的建设进度，确保今年新引进资金１２亿元，新建工业厂房５０万平方米。切实抓好分宜城西工业园和渝水良山工业园建设，鼓励工业企业退城进园，积极支持新钢、新电、赣西供电局利用自身优势到工业园区创办特色工业园。争取通过３年努力，使分宜城西工业园、渝水良山工业园成为我省首批年销售收入２０～５０亿元的县级工业园区。加快建成双林以夏布加工为主和下村以非金属矿产资源精深加工为主等一批特色工业重镇。

（三）创建国家园林城市，加快建设一流的人居环境

积极实施“三城”创建工程，打造现代生态园林城市。坚持规划先行，在总规修编的基础上，搞好分区规划、片区规划，做好控制性、修建性详细规划的编制，力争用两年时间，全面完成与中心城市相适应的规划体系。全面推进城市净化、美化、亮化、绿化。切实抓好１３条背街小巷的综合整治，加快城南旧城改造步伐，支持新钢公司改造厂容厂貌；继续抓好城区主干道“穿衣戴帽”工程；抓紧城郊水源涵养林和环城绿化带建设，高标准组织单位庭院绿化美化整体改造；加快建成仙来东大道、校园路、清宜公路西段等标志性城市进出口景观。大力实施城市建筑组群开发，搞好组群搭配和造型设计，加快建设一批以高层建筑为主体的建筑群体，高水平建成奥克斯皇家花园等一批现代化住宅小区。高起点、高标准组织实施孔目江新城区建设工程，整体推进孔目江改造，力争用三年时间，基本建成孔目江新城区。

切实加强城市基础设施建设，着力增强城市的承载能力。进一步完善主城区路网体系，继续抓好仙女湖大道、高新区南一横路和中山中路等道路建设，全面完成冶金西路、科环路、劳动路立交桥北匝道等７条城市道路，开工兴建白竹路、孔目江沿江路、长青南路及立交桥、天工大道及连接线、人民北路及立交涵等城市路桥和高新区北二横等６条道路。切实抓好城市管网改造，积极启动城市燃气管网整体改造和城东燃气工程，加快城市排水排污管网建设。积极组织实施城市饮用水改善工程，全面完成城南供水管网改造，加紧实施仙女湖区供水扩建工程和城市供水管网的整体建设改造，着力做好白云水库立项、设计、招商工作，取消城市自备生活水源，确保到２００７年，主城区生活饮用水供水能力达到５０万人口规模，城市饮用水质量得到明显提升。继续做好城东污水处理厂、驴脚岭垃圾处理场等项目续建，加快启动城市防洪四期工程。抓紧实施城南、城北汽车站整体搬迁，配合做好浙赣铁路电气化改造和武吉高速公路建设相关工作。高起点建设唯滕休闲中心、春龙国际大酒店等一批宾馆酒楼。

加快商贸物流和旅游设施建设，着力提升中心城市的发展人气。大力实施现代物流体系建设工程，继续抓好高新区时代广尝九鼎汽车大市场钢材大市场等一批专业市场续建工程，开工兴建高新区中国商贸城、诺玛特大型仓储式超市、东方巴黎购物广场等综合市场，加快市级商业中心、四个区域性商业闹区建设步伐，切实抓好胜利路、城北人民广场等城市闹区改造，抓紧洪客隆商业步行街开工兴建。继续抓好公路货运中心、铁路货运中心建设，加快建成赣西最大的现代物流中心。切实抓好新余展示馆建设。大力组织实施仙女湖旅游城和抱石名园建设工程，积极创建中国优秀旅游城市和仙女湖国家４Ａ级旅游区，加快干宝路、旅游环城公路建设，积极规划论证天工开物城、东方丽人城等项目开发。力争用三年时间，全面完成抱石公园改造，加快建成仙女湖旅游城。

规范发展房地产业，着力推进城市经营管理的体制创新。建立健全建设、国土、房管等部门联审机制，鼓励成片开发，严控零星开发，科学编制房地产中长期发展规划和年度用地计划，适时适度搞好房地产的市场投放，严格按规定征收各项规费。实施鼓励住房消费的积极政策，加大住房公积金归集、发放力度，不断增强居民换购住房的支付能力。加快建成房地产交易市场，鼓励存量住房上市交易，大力盘活房地产二、三级市常按照“管、保分离”的原则，公开拍卖城区道路保洁权和园林、市政养护作业权。整合城市资源，加强规划监管，严格规划设计审批，对城中村农民建房实行每季度集中审批一次，全面清理城市违章建筑，切实解决乱搭乱建、违章建筑问题。实施畅通工程，完善公交线路，规范整治“五车”（摩的、拐的、出租车、黄包车、板车），完成城东公交场站建设，抓紧通济停车场青年路停车场建设和城市立交设施的规划兴建，加快形成便捷畅通的公交循环体系。严格施工现场管理，推行全封闭文明施工，切实解决建筑施工扰民和影响市容环境问题。以创建全国社区建设先进市为目标，大力推进城市划片整治，合理确定社区规模，加快事权下移步伐，强化社区服务功能，加快建立市、区、街道、居委会四级管理网络。

（四）加强农业经济结构战略性调整，加速推进农业产业化和农村城镇化

全面落实党的惠农政策，进一步提高农业综合生产能力。坚持“多予、少娶放活”的方针，进一步稳定、完善和强化各项支农政策，切实做到已经实行的政策不变，已给农民的实惠不减，支农惠农的力度只能加大、不能减弱。从今年起，全市一律免征农业税，实现农民合同内“零”负担；继续实施粮食直补、良种补贴和农机具购置补贴，继续实施粮食最低收购价政策，确保农民利益不受损害。实行最严格的耕地保护制度，切实抓好耕地占补平衡，确保基本农田保有率巩固在８５．７％的水平上。实施良种良法工程，集中力量建设一批优质粮食基地。认真抓好粮食批发市场建设，加快直属粮库异地改建步伐，组织好粮食购销合同签订工作，千方百计调动和保护好农民种粮积极性。

突出抓好农业经济结构战略性调整，加快形成以特色产业开发主导农村经济发展的新格局。坚持以基地开发促进产业开发，以产业带中心乡镇建设支撑特色产业快速发展。围绕培育壮大草食畜禽、花卉苗木、优质苎麻、新余蜜桔等四大高效特色产业，大力发展优质、高产、高效、生态、安全农业，抓紧实施８０万头生猪、１０万头肉牛、５万亩花卉苗木、５万亩新余蜜桔、５万亩优质苎麻等重大农业项目，切实抓好商品蔬菜基地开发，积极推进优势农产品产业带建设，加强特色产业重镇培育，加快形成“一县一业、数乡一品”的发展格局。着手启动“十百千示范工程”，高标准建设１０个现代农业示范园区和１００个生态畜牧小区，着力培养１０００户农村双带致富示范户。

大力培育龙头企业，不断提升农业产业化经营水平。坚持把龙头企业培育作为产业化核心来抓，积极支持现有龙头企业做大做强，加快引进、培育一批产业化龙头企业。高标准建成百乐粮食工业园和分宜夏布城。大力实施品牌战略，精心培育一批知名农业品牌。切实加强农村专业合作经济组织建设，重点培植１００户农产品经纪人和营销大户。大力实施“无公害食品行动计划”，积极推行标准化生产，抓好一批无公害农产品基地和绿色食品基地建设。健全市、县（区）两级质检机构，加快建立完善农副产品质量认证体系、检验检测体系和动植物重大疫病防控体系。

着力组织实施小康文明村镇建设工程，全面改善农村生产生活条件。完善村镇规划体系，强化建筑设计管理，规范村民建房行为，严禁未批先建、占用基本农田建房等现象；强化镇村功能设施建设，逐步完成农村改水、改厕、改路和电网改造，实施组组通公路和村村通客车、信息、广播电视工程；按照“坚强的领导班子、高效的主导产业、优美的生活环境、良好的社会秩序”的要求，积极开展小康红旗村镇创建活动，高标准推进以１０个中心镇、１００个中心村为主的小康文明村镇建设，力争三年内农村面貌发生根本改观。切实抓好农田水利建设，加快建成一批高标准基本农田，重点实施好袁惠渠续建配套与节水改造项目和彰湖、鹄山水库除险加固二期工程，基本完成３０座病险水库加固任务。着力抓好渝水区经济林、分宜县生态林、仙女湖生态公益林建设，积极争取国家、省级生态公益林补助项目，启动市级生态公益林项目，全面实施日本政府贷款江西造林项目，加快建设笋竹两用林、高效生态型油茶和工业原料林等三大基地。

积极组织实施十万农民培训工程，加速农民转移就业。着眼于增强农民就业、致富能力，切实抓好农民培训工作，加快建立土地征用每亩增加１０００元列入农民培训基金的经费征集制度，建立统一培训、资金专款集中使用的培训和管理机制。以技能实用和保证就业为前提，面向民办职业院校实行农民培训公开招标。力争三年左右培训农民１０万人以上，今年培训农民３．４万人次。

（五）积极发展各项社会事业，促进人的全面发展

认真实施“科教兴市”战略，全面加快文化、广电、卫生、体育等各项事业发展。坚持教育优先发展的战略地位，进一步落实基础教育以县为主的管理体制，统筹解决农村中小学建设和教师工资发放问题，提高农村中小学义务教育公用经费补助标准。继续抓好高专专升本工作和市一中、四中、十中、分宜中学等优质高中建设，积极启动长青育才学校建设工程。大力实施高中阶段教育普及工程，制定完善全市普及高中教育规划，优化网点布局，充实师资力量，加强学校建设，力争通过三年奋斗，率先在全省普及高中阶段教育。以创建全国职业教育示范市为目标，切实抓好现代职业教育基地建设工程的组织实施，加快职业教育片区建设，大力兴办一批示范性职业院校和特色专业；设立民办教育发展信用担保基金和专项经费，规划启动职业教育实训基地建设，全力支持渝工、中山、赣西等知名院校快速发展。力争到２００７年，职业院校在校学生总规模达到１０万人以上。积极推进科技创新体系建设，引进技术、人才和成果，加强科技合作与行业科技攻关，广泛开展科研成果和技术创新项目推介，抓紧组织实施一批重大科技项目，加快重点企业信息化改造，力争科技进步综合排名进入全省前三位。切实加快市科技馆建设，搞好公众科技素养调查，广泛开展科普示范社区、示范学校和科普致富示范村创建活动，争取三年内建成全省科普教育示范城。积极启动市文化艺术产业中心建设，加快文化大市场文化影视城建设步伐，进一步完善市？？物馆、珍宝馆、图书馆、档案馆和农村基层文化设施，抓好广场文化、社区文化建设，广泛开展群众性文化活动；大力加强文化、出版物市场监管；以参加第三届江西省艺术节为契机，实施精品战略，努力推出更多的文化艺术精品力作。积极推进广播电视数字化改造。高起点做好第十二届省运会各项筹备、备战工作，加快文化健身广场建设，积极抓好全民健身中心申报立项建设工作，广泛开展全民健身运动。改善镇、村医疗卫生条件，健全农村药品供应网点，着力抓好村级卫生所标准化建设；继续抓好新型农村合作医疗试点工作。整合医疗资源，抓紧分离企业办医院；加快专病专科建设，提升医疗服务品牌；切实加强公共卫生体系、食品和药品安全信用体系建设，加快实施市疾控中心、传染病专科医院、医疗救治中心三大国债项目和市妇幼保健院改造扩建。强化公共安全事件预警处置机制，坚决防止重大公共安全事故发生。加强军事斗争准备，进一步做好国防动员和民兵预备役工作，动员全市军民扎实推进“双拥”工作，确保实现全国双拥模范城五连冠。

大力实施可持续发展战略，统筹推进人与自然的和谐发展。切实加强环境建设，开工兴建市环保监测监察指挥中心；大力实施城市大气污染、水系污染、噪声污染防治等重点环保工程，实现空气质量每日一报目标；加快仙女湖水源涵养地及生物多样性功能保护区建设；加紧实施袁惠渠水质污染综合整治，争取用２～３年的时间，使袁惠渠水质尽快达到Ш类标准。依法保护和合理利用矿产资源，积极推行探矿权、采矿权的统一规划、管理和拍卖。做好土地利用总体规划修编工作，强化土地整理；切实加大土地开发调控力度，严格落实土地管理各项制度，完善土地征收征用程序和补偿机制，促进土地利用向集约化方向发展。积极探索新形势下人口与计划生育工作的新思路、新途径，创新工作机制，推进综合改革，搞好优质服务，落实利益导向政策，完善村（居）民自治组织，突出抓好特殊群体的计划生育管理，下力气综合治理出生人口性别结构，稳定低生育水平，确保计划生育工作继续走在全省前列。进一步做好妇儿、老龄和关心下一代工作，切实维护妇女和未成年人、老年人、残疾人的合法权益。抓好经济普查。精心组织编制第十一个五年规划。

（六）高度关注人民群众的切身利益，加快构建和谐平安新余

切实抓好就业大市场建设，加快构建城乡一体的就业体系。实施增加就业岗位工程，大力发展中小企业和劳动密集型产业，加强再就业基地和非正规劳动组织建设，抓好高新区和工业园区用工配置，积极开发社区服务和公益性就业岗位；健全帮扶机制，积极举办就业招聘洽谈大会和“４０５０”人员专场招聘会，重点帮助下岗失业人员、“４０５０”人员和困难人群实现再就业；实施积极的就业政策，全面落实小额贷款、社保补贴、税费减免等各项再就业扶持政策；着力抓好就业大市场建设，积极开辟面向沿海的就业再就业绿色通道，争取两年左右把就业大市场建成集就业、社会保障、职业培训、技能鉴定、人力资源开发于一体的区域性中心市场；强化社区就业服务和劳动保障平台建设，抓好观念引导、职业介绍与就业培训，推行劳务派遣和劳动保障事业代理，加快建立有利于扩大就业的目标责任体系、劳务输出基地体系、就业政策体系、就业服务体系、就业培训体系、执法维权体系。力争全年新增城镇就业２万人，下岗？？？工再就业６０００人，安置“４０５０”人员再就业１１００人，省外劳务输出１６．５万人。

大力发展社会保障事业，加快建立与经济发展水平相适应的社会保障体系。切实加大保费征缴力度，积极做好改制企业职工社会保障关系接续工作，加快下岗职工基本生活保障向失业保险并轨，确保下岗出中心职工基本生活费和企业离退休人员养老金按时足额发放，保障改制破产企业职工和国有企业新裁减人员基本生活。积极做好扩保工作，加快建立机关和事业单位社会保障制度，尽快将非公有制经济从业人员养老、医疗、失业保险等统一纳入社会保障体系建设。完善城乡最低生活保障制度，将符合条件的城乡困难群体全部纳入保障范围。积极探索建立社会保险、社会救助、社会福利和慈善事业相互衔接，养老保险、医疗保险、失业保险、工伤保险、生育保险一体征缴的社会保障体系。

以人为本关注民生，切实解决人民群众的实际困难。完善社会救助体系，建立扶贫济困基金，开展扶贫帮困活动，着力解决好贫困群众在就医、子女入学升学、居住等方面的实际困难。完善农村“五保”和城镇“三无”对象集中供养制度。继续开展拖欠工程款和农民工工资专项整治，认真落实城乡建设中征地拆迁补偿安置政策，切实保障失地农民和城市拆迁户的合法权益。全面实施与完善城市价格收费公示和涉农收费公示制度，加大对农资价格和教育、医疗、住宅、电力、交通、旅游等的收费专项检查，深入开展中介服务费、经营服务性价格等的清理、整治和规范工作。妥善做好江口库区移民工作，努力改善移民生产生活条件。

大力实施依法治市战略，积极推进和谐平安新余创建工作。坚持把创建和谐平安新余作为重要的城市品牌来抓，强化防控体系，完善预警机制，优化警力配置，切实加强对重点地区、重点行业、重点人口、重要时期的治安管理，保持严打高压态势，严厉打击各种刑事犯罪活动；做好“四五”普法验收工作，完善法律援助制度，全面完成司法所用房和规范化建设，依法加强宗教事务管理，积极预防和妥善处置群体性、突发性事件；维护国家安全，确保社会政治稳定，努力争创全国社会治安综合治理先进市。畅通信访渠道，清理信访积案，认真做好群众来信来访和疏导教育工作，切实解决好人民群众关注的热点难点问题。全面实施安全生产许可制度，建立健全安全生产监管体系、应急救援体系和以企业为主体的责任体系，积极开展安全生产专项整治，强化重点行业、重点领域、重点危险源的日常监控，抓好消防安全、森林防火工作，努力杜绝重特大安全事故的发生。

三、切实加强政府自身建设

继续贯彻落实国家宏观调控政策，不断增强破解难题、统筹发展的能力。各级政府和领导干部要坚持用科学发展观统领经济社会发展的全局，更加注重转变经济增长方式，更加注重统筹城乡发展，更加注重维护和实现社会公平，更加注重加强生态建设和环境保护，更加注重促进社会主义物质文明、政治文明、精神文明的协调发展，努力把对科学发展观的认识成果转化为促进发展的正确思路，转化为实现全面、协调、可持续发展的体制机制，转化为领导改革开放和现代化建设的实际能力。要进一步巩固宏观调控已取得的成果，切实加强土地管理，搞好煤、电、油、运综合协调，高度关注和解决好影响经济发展的经济结构不优、体制机制不活、瓶？？？制约突出、基础设施建设滞后等问题，统筹推进城乡经济和社会事业协调发展。

切实加强社会主义政治文明建设，不断提高依法行政水平。坚持依法行政，规范行政许可，完善行政执法评议考核制和过错责任追究制度，加快建立权责明确、行为规范、监督有效、保障有力的行政执法机制。强化宪法意识，认真办理人大代表、政协委员的提案和建议，积极加强与民主党派、工商联、无党派人士和各人民团体的联系，支持工会、共青团、妇联等群众性组织开展工作。改进决策方式，完善决策机制，建立健全人民群众建议征集制度和重大事项集体决策、公示听证、专家咨询制度。发展电子政务，推进网上办公。扩大基层民主，全面开展农村民主法制示范村创建和法律进社区活动。切实抓好领导干部廉洁自律、查办大案要案、纠正部门和行业不正之风工作，继续完善国库集中支付、会计集中核算、政府采购和土地出让、工程建设招投标制度，加强领导干部任期经济责任审计、财务审计、绩效审计工作，实行多种形式述职述廉，健全重大事务报告和政务环境评议评价制度，建立健全教育、制度、监督并重的惩治和预防腐败体系。

大力加强政府效能建设，不断改进政府机关作风。各级政府和领导干部务必认真实践“三个代表”重要思想，以开展保持共产党员先进性教育活动为契机，切实增强中心意识和大局意识，不折不扣地落实好中央和省委、省政府的政策措施，不折不扣地贯彻好市委、市政府的决策部署，着力解决有令不行、有禁不止的问题；积极组织开展“加强机关作风建设，优化经济发展环境”综合测评活动，全面推行“末位诫勉制”和“末位淘汰制”；进一步健全抓落实的责任机制和工作机制，完善科学目标管理考核制度，切实抓好各项工作尤其是重大决策部署的全程跟踪督查。要进一步增强创新意识、服务意识、学习意识，真正把政府工作重点转变到提供公共产品、公共服务上来，把全体市民的关注点转移到创新、创业上来，广泛开展各类学习型组织创建活动，加快建立鼓励学习、鼓励创新、鼓励创业的激励机制。要大力实施人才战略，建立完善人才市场体系和评价体系，加快建立鼓励人才脱颖而出的用人机制，努力建设学习型、服务型、创新型政府。

各位代表，加快区域中心城市建设，率先实现全面小康，任务艰巨，使命光荣，需要全市上下同心协力，发奋拼搏，坚定不移地朝着既定目标开拓前进！让我们紧密团结在以胡锦涛为总书记的党中央周围，在省委、省政府和市委的正确领导下，把时代的召唤当作前进的动力，把崇高的使命化为积极的行动，以更加创新的思维、勇于率先的胆识、扎实苦干的作风，全面做好今年的各项工作，推动我市经济社会发展实现新的跨越！
